# 3D Measurement Simulation and Relative Pointing Error Verification of the Telescope Mount Assembly Subsystem for the Large Synoptic Survey Telescope †

**DOI:** 10.3390/s18093023

**Published:** 2018-09-10

**Authors:** Unai Mutilba, Gorka Kortaberria, Fernando Egaña, Jose Antonio Yagüe-Fabra

**Affiliations:** 1 Department of Mechanical Engineering, IK4-Tekniker, 20600 Eibar, Spain; gorka.kortaberria@tekniker.es (G.K.); fernando.egana@tekniker.es (F.E.); 2 I3A, University of Zaragoza, 50018 Zaragoza, Spain; jyague@unizar.es

**Keywords:** RPE, large synoptic survey telescope (LSST), telescope mount assembly (TMA), laser tracker, simulation, active alignment system, mirror positioning

## Abstract

An engineering validation of a large optical telescope consists of executing major performing tests at the subsystem level to verify the overall engineering performance of the observatory. Thus, the relative pointing error verification of the telescope mount assembly subsystem is of special interest to guarantee the absolute pointing performance of the large synoptic survey telescope. This paper presents a new verification method for the relative pointing error assessment of the telescope mount assembly, based on laser tracker technology and several fiducial points fixed to the floor. Monte-Carlo-based simulation results show that the presented methodology is fit for purpose, even if floor movement occurs due to temperature variation during the measurement acquisition process. A further research about laser tracker technology integration into the telescope structure may suggest that such laser tracker technology could be permanently installed in the telescope in order to provide an active alignment system that aims to detect and correct possible misalignment between mirrors or to provide the required mirror positioning verification accuracy after maintenance activities. The obtained results show that two on-board laser tracker systems combined with eight measurement targets could result in measurement uncertainties that are better than 1 arcsec, which would provide a reliable built-in metrology tool for large telescopes.

## 1. Introduction

The Large Synoptic Survey Telescope (LSST) is a large (8.4 m) wide-field (3.5 degree) survey telescope, which will be located on the summit of Cerro Pachón in Chile. The Telescope Mount Assembly (TMA) subsystem points at and tracks fields on the sky, by providing motions about the azimuth and elevation axes. Therefore, it provides pointing, tracking, and slewing system performance requirements to comply with the space survey mission [1]. TMA is currently being assembled in the north of Spain, and the presented method will assess the relative pointing error (RPE) of the subsystem [2].

When observing the sky, it is of great interest to make sure that the telescope is pointing towards the intended location on the sky as accurately as possible, to ensure that it is pointed towards the correct target source and, consequently, to use accurate photometric and astrometric information that is related to that target. To get an overview, while an amateur telescope can reasonably aspire to 30 arcsec, a giant observatory instrument such as the LSST can point to an absolute pointing performance of 1 arcsec [3].

It shall be highlighted that the pointing and alignment performance of the LSST will have a very strong influence on the quality of the scientific results obtainable. There are typically four requirements that are of particular interest on a large telescope for scientists: The absolute pointing error (APE), is defined as the angular separation between the actual direction and the intended telescope line-of-sight. The absolute measurement accuracy (AMA) is defined as the angular separation between the actual direction and the reconstructed direction of the telescope. The absolute pointing drift (APD), defined as the change in the angular separation between the actual direction and the intended direction of the telescope over the observation time. The relative pointing error (RPE) is defined as the angular separation between the actual direction of an axis and a reference axis, over the instrument exposure time [4] and, therefore, it supplies the short term stability for a large telescope. The RPE requirement for the TMA subsystem in the LSST project is limited to 50 arcsec. at a 95% confidence level (*k* = 2) [5].

In this scenario, no telescope structures and control systems are perfect, so the pointing error always exists. Pointing errors include repeatable and non-repeatable errors. Most of the pointing errors are repeatable, and there are different methods to verify those errors and to provide geometric compensation information to the pointing calibration models on the telescope’s final working location [6,7]. However, an end-to-end test of the complete LSST, in order to check the pointing performance and the correct alignment of all the elements, is not possible until the final assembly in Chile is complete. For this reason, the TMA subsystem will first be tested, including RPE requirement, at the factory with surrogate masses, to replace the optical payloads with the aim of avoiding ‘late surprises’ during the LSST construction in Chile. Thus, LSST project requires a new RPE verification method based on laser tracker technology for the engineering validation of the TMA subsystem.

The main way to quantify the absolute pointing error of a telescope is assessed by the “star tracker” method [8]. As star positions in the sky are known with very high precision, the identification of the star signals provides a powerful tool to check the absolute telescope alignment. In practice, guide star catalogue is considered as a measurable point cloud on the sky, and the least square fitting technique provides the solution for the absolute pointing error assessment [9].

There are different techniques to test the RPE of a critical subsystem in a telescope: (a) To employ an optical pointing telescope (OPT) mounted on the subsystem under verification. The pointing verification measurements of the Atacama large millimetre/submillimetre array (ALMA) antenna were performed using an OPT mounted on the antenna backup structure [10]; (b) error budget is also another approach to make error allocations to the relevant subsystems, i.e., structure, thermal, instrument. Thus, a calibration campaign allows verification at an early stage in the programme, when the main parameters of the optical system are within the allocated alignment budget [4]; (c) laser tracker technology that has been previously discussed for defining coordinates for aligning optical systems [11,12] and also have been considered as a built-in alignment tool for various new-generation large telescope projects, such as the Large Binocular Telescope (LBT) [13] and the Extremely Large Telescope (ELT). In this scenario, the LSST project has defined laser tracker technology as a “facility alignment system”, and it will be used during the integration to monitor and to position the elements. Additionally, mount pointing and alignment testing will be done on-site before installing the mirrors and the camera with laser tracker technology, using surrogate masses and a small alignment telescope mounted onto the mount structure [14].

Besides the RPE assessment exercise, large telescopes also require on-board 3D metrology systems to align optical mirrors, or to provide an active alignment system in order to allow misalignment correction between optics, either when they deflect due to variations in thermal environment or when gravity-induced structural flexure affects to the mount [15]. Rakich et al. suggest a telescope metrology system (TMS) that incorporates a large number of absolute distance-measuring interferometers to detect misalignments between primary and secondary mirrors for the giant Magellan telescope (GMT) [15]. Laser tracker technology has also been under research for that purpose, as commented by Sandwith et al. [16] for the LSST. Rakich et al. also suggested to employ a laser tracker for the active alignment of the large binocular telescope (LBT) [13].

In this paper, a new measurement methodology is presented for the RPE assessment of the TMA for any motion that is within the pointing range of the LSST, based on laser tracker technology and fiducial points. In addition, a Monte-Carlo based simulation platform has been built to assess the achievable accuracy with an on-board laser tracker system, either for active alignment or for the mirror positioning activities at the LSST. The aim of the presented research work is to further investigate the application of laser tracker technology on large scale telescopes, and to provide reliable measurement strategies to be compatible with the required mirror positioning accuracy, which is limited to 1 ÷ 5 arcsec for large telescopes [3].

## 2. Relative Pointing Error Verification

A detailed description of the developed RPE verification method for the LSST is presented.

### 2.1. Measurement Scenario

The optical axis of the LSST is defined at the M1M3 primary/tertiary mirrors, so that one of the limitations tackled by any RPE measurement procedure is the measurement of the M1M3 mirrors for any pointing motion within a LSST pointing range. Additionally, all the performance requirements must be met for the observing angles between 15 and 86.5 degrees for elevation angles, and from 0 to 360 degrees for azimuth angles. However for maintenance work, the TMA should be able to point from horizon to zenith (i.e., elevation angles from 0 to 90 degrees) [5].

**Figure 1:**
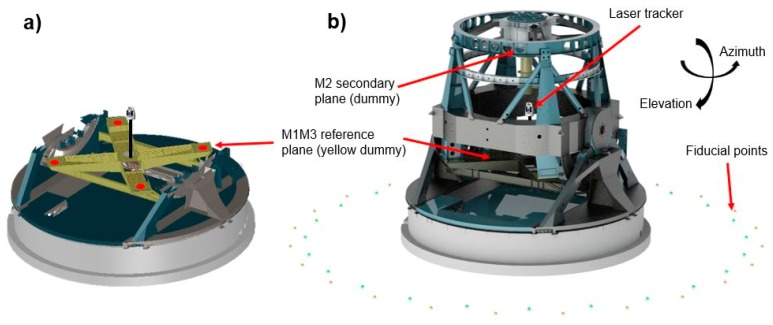
Measurement scenario for RPE assessment. (**a**) M1M3 measurement plane (measurement targets in red) (**b**) Complete LSST measurement scenario.

The measurement of the LSST telescope was a large-scale metrology (LSM) exercise [17], since the dimension of the measurement scenario was up to 40 m of diameter. Thus, LSM technology was employed for the suggested RPE characterization: The Leica AT402 laser tracker technology combined with Spatial Analyzer (SA) software from New Rivers Kinematics. Figure 1 shows the measurement scenario for the RPE assessment. Figure 1b illustrates the complete measurement scenario. Figure 1a shows the M1M3 measurement plane, where the measurement targets are depicted in red. Additionally, it also shows that the engineering validation at the subsystem level was verified with dummies instead of the real optical elements.

**Figure 2:**
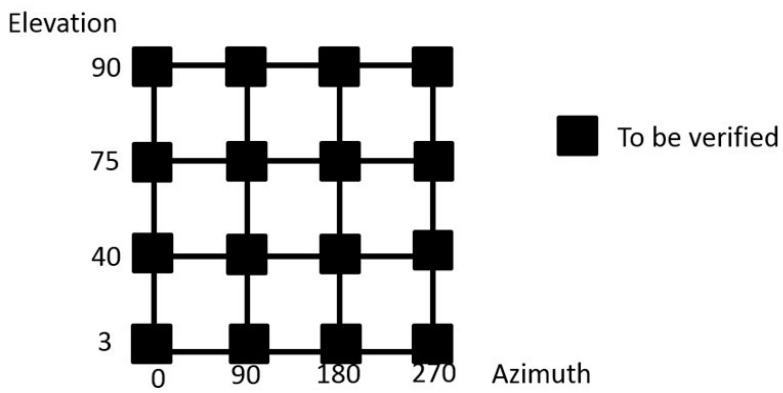
Mapping matrix for the RPE test.

In this measurement scenario, a pointing matrix was defined to characterize the RPE measurement test of the TMA within the pointing range of the LSST. Four elevation angles at four different azimuth positions were defined to represent any pointing direction on the sky, as shown in Figure 2.

### 2.2. Measurement Procedure

A new measurement procedure for the RPE assessment was defined as follows: A laser tracker was placed inside of the LSST, close to its origin, and a metrology network comprising a reference point cloud was fixed to the floor, outside and surrounding the LSST telescope. This metrology network was of special importance, as any laser tracker location during the whole measurement process was solved by the measurement of this fiducial metrology network. Thus, the RPE measurement procedure consisted of measurements of the metrology network to locate the laser tracker, and afterwards, measurements of the optical axis of the TMA by measuring four target points at the M1M3 reference plane. Thus, by locating the M1M3 reference plane on an earth fixed reference system, i.e., the floor, each observation angle for TMA was characterized and compared to the input position, which means the RPE assessment. The measurement process was repeated for each of the pointing positions defined in Figure 2, and within the pointing range of the LSST [2]. It should be highlighted that laser tracker position was fixed close to the LSST origin, to nullify the range of the angle of incidence from the laser tracker to the reflectors. Thus, the laser tracker tilts with the rotation centre of the telescope and incidence angle does not change which means that it will not cause a longer travelling path of the beam inside the prism. For the LSST project, 25 mm hollow corner cube optics were employed.

An accurate reference point cloud comprised of 48 points was defined on the floor outside and surrounding the telescope. Twenty-four points created a 15 m radius circle, and 24 additional points defined a 16 m radius with an offset of 7.5° to the previous one. Its circular shape optimized the visibility challenge for any azimuth-pointing position of the telescope. Additionally, those points were fixed to the floor, minimizing thermal gradients effects. Figure 1 shows the metrology network arrangement around the LSST and the M1M3 reference plane where every measurement shall be executed.

**Figure 3:**
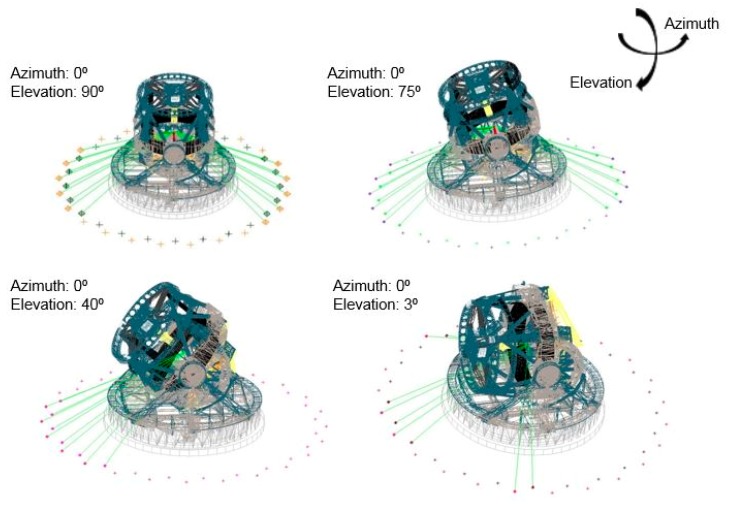
Visibility study overview from inside placed laser tracker, from the zenith to the horizon pointing direction.

The biggest challenge to meet the RPE measurement specification is to ensure that the line of sight between the M1M3 reference plane and the metrology network for any pointing position. Thus, a visibility study was executed within SA software for any elevation axis position. Figure 3 visually represents the line of sight for any elevation angle of the TMA.

Green lines in Figure 3 show the line of sight from the inside-placed laser tracker to the points that comprise the fiducial metrology network. The visibility became worse from the zenith to the horizon TMA pointing direction. However, any TMA pointing direction could be assessed by the presented measurement procedure.

### 2.3. Measurement Simulation

A simulation model, based on the Monte-Carlo technique, was developed to assess the RPE measurement uncertainty according to the developed measurement procedure. The simulation model was developed within SA software, so a commercial tool was employed to code the simulation model [18]. For that simulation, a Gaussian random number generator (utilizing a Box-Muller algorithm) mathematically simulates the measuring scenario with 500 sensitivity samples [19], and the standard deviation parameter was calculated as an uncertainty indicator of the simulated measurement methodology. In addition, the Box-Muller algorithm executed a laser tracker error model according to the specifications of the laser tracker’s manufacturer (Leica) at a 68.3% confidence level (*k* = 1), where:

(a) U_F_ = Uncertainty of the fixed length error that applies to all distance measurements. For a Leica AT402 laser tracker, it is 0.00762 mm.
(b) U_M_ = Uncertainty of the additional length error as the measurement distance increases. For a Leica AT402 laser tracker it is 2.5 µm/m.
(c) U_A_ = Uncertainty of angle measurements. For a Leica AT402 laser tracker it is 1 arcsec.

The RPE measurement simulation process had two main stages: The first stage was executed to characterize the reference metrology network, and the second stage aims to quantified the measurement uncertainty on the RPE assessment.

#### 2.3.1. Metrology Network Characterization

The unified spatial metrology network (USMN) tool was employed for coordinate uncertainty field computation [18]. The fundamentals of this technique were that the uncertainty of a particular measurement was simulated using the knowledge of the position of the measurement instrument and the non-isotropic uncertainty of the instrument. The best fitting of all points was weighted, giving less weight to coordinates with higher uncertainty. Since the uncertainty of measurements taken using a laser tracker is known to be considerably better in range than in angle, the distance measurements were given a greater weight than the angle-derived measurements. The end result of this approach was therefore similar to multilateration. It is not however pure multilateration, since the angle-derived measurements are still used to some extent [20].

For the metrology network characterization, the laser tracker was fixed to the TMA, as an on-board 3D metrology system and the 16 TMA pointing positions were performed. Therefore, every fiducial point was measured from different laser tracker locations, which allowed the characterization of the position of every fiducial point that was fixed to the floor and that was relative to the TMA. The simulation is executed with 500 samples, according to the Leica AT402 laser tracker error model, and the standard deviation parameter of every fiducial point coordinate on each axis direction was obtained from the simulation. Thus, the expanded uncertainty of every fiducial point on each axis direction was obtained by multiplying the standard deviation times the coverage factor (*k*):

(1) $$U_{x}=k\times s_{x};\;U_{y}=k\times s_{y};\;U_{z}=k\times s_{z}$$

where:

- *k* = coverage factor
- *s* = standard deviation

**Figure 4:**
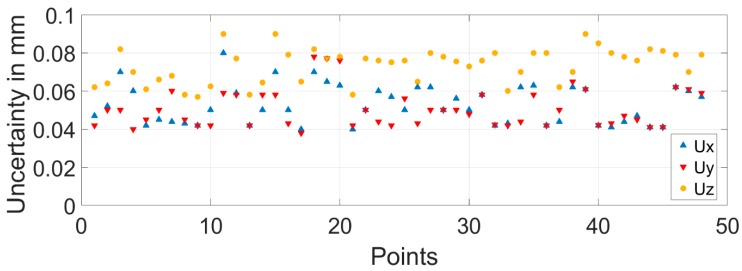
Measurement uncertainty for metrology network characterization.

According to the executed simulation, every point uncertainty was better than 0.1 mm for a 95% confidence level (*k* = 2), as shown in Figure 4.

The simulation result correlated with the research of Rakich et al. at the LBT active alignment system with laser tracker technology [13].

#### 2.3.2. RPE Measurement Simulation

**Figure 5:**
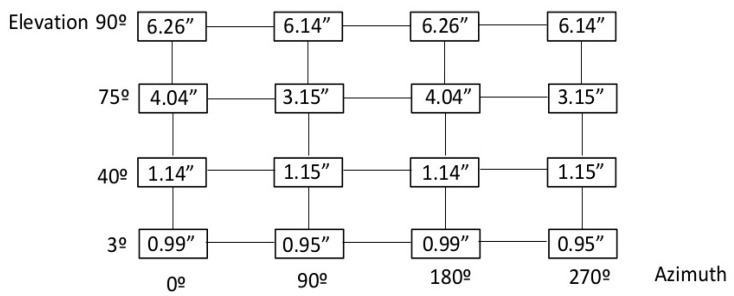
RPE measurement uncertainty results (in arcseconds).

Once checked that the metrology network allowed the location of the laser tracker at any measurement position within the measurement scenario; in fact any TMA pointing direction could be assessed within the pointing range of the LSST. Thus, the M1M3 reference plane was measured and referenced to the earth-fixed reference system, so that the TMA pointing direction was accurately measured for any pointing direction on the sky. For practical issues, the pointing range of the TMA is discretised as shown in Figure 2. Figure 5 shows the RPE measurement uncertainty results, for a 95% confidence level (*k* = 2), obtained by the Monte-Carlo simulation according to the mapping matrix represented in Figure 2.

Simulation results showed that uncertainty values were higher when TMA was pointing to the zenith (elevation angle equals 90 degrees). At this pointing position, the normal vector of the M1M3 plane perfectly followed the “z” direction or zenith and, therefore, the uncertainty value was the largest. As the M1M3 plane moved away from the zenith to the horizon, the uncertainty value became smaller.

This means that, for elevation angles near to the zenith pointing position, the plane-based post-processing method showed the highest uncertainty values and, therefore, it seemed that measurement results became worse because of the employed post-processing method. To improve those results, a best-fit based post-processing method was analysed for both 75 degree and 90 degree elevation angles.

While the plane-based post-processing method decomposed the plane vector into 6 degrees of freedom to obtain the rotation angles around the zenith and the elevation axis of the LSST, the best-fit based post-processing method matched the M1M3 points, measured at 75 degree and 90 degree pointing positions respectively, with the M1M3 points measured at the origin, the azimuth at 0 degrees, and pointing to the zenith. The obtained results improved the uncertainty values down to 1 ÷ 2 arcsec for every pointing position of the LSST, which meant that the presented new verification procedure dealt with the RPE requirement, limited to 50 arcsec in a temperature stable measurement scenario (e.g., 20 ± 1 °C on the complete LSST volume).

It is likely that the best-fit based post-processing method will be implemented into the future real measurement scenario, since it could take into account and cope with the real flatness of the mirrors. However, in the current simulation case, the real geometry of the plane did not affect the measurement uncertainty of the RPE assessment.

**Figure 6:**
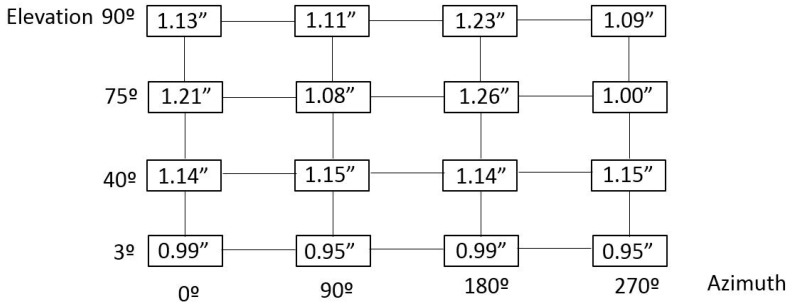
Improved RPE measurement uncertainty results (in arcseconds).

Figure 6 shows the RPE measurement uncertainty results for a 95% confidence level (*k* = 2), combining plane-based post-processing and best-fit based post-processing methods.

### 2.4. Floor Movement

Previous simulations consider an absolute and fixed metrology network, but the floor suffers from dimensional drift due to the ambient temperature variation. Therefore, a more realistic simulation was executed to determine the fit for purpose for the presented measurement methodology.

The aim at this point was to quantify the 3D movement of the floor, where fiducial points are fixed, so a 24 h measurement was executed on the premises where TMA was being assembled, in the north of Spain. Results provided a more realistic overview of how floor moved, so a new simulation is performed, considering the floor movement as an input variable for the simulation.

**Figure 7:**
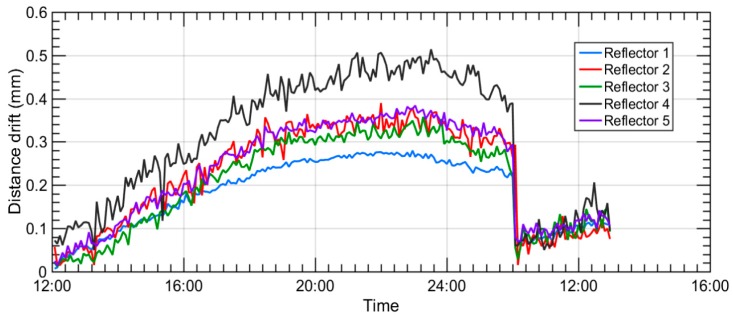
Floor movement assessment for the TMA RPE assessment.

The measurement of the floor was executed with a Leica AT402 laser tracker and five measurement reflectors. They were repeatedly measured, every 5 min, for one day. It was assumed that the laser tracker was fixed to the floor, and that measurement points moved according to the floor drift. Thus, Figure 7 represents the position drift for the five points as the variation of the distance from each of those measurement points to the fixed laser tracker. Measurement results showed that the dimensional drift of every measurement point was within 0.5 mm, for a temperature change of 4 °C. Figure 7 depicts the floor movement assessment for the premises where TMA was being mounted.

The automatic data acquisition process was interrupted during the first night due to unknown reasons. It was reactivated next morning, and the dimensional drift curves depicted in Figure 7 show that the second morning floor deviation values were similar to the first morning acquired values.

**Figure 8:**
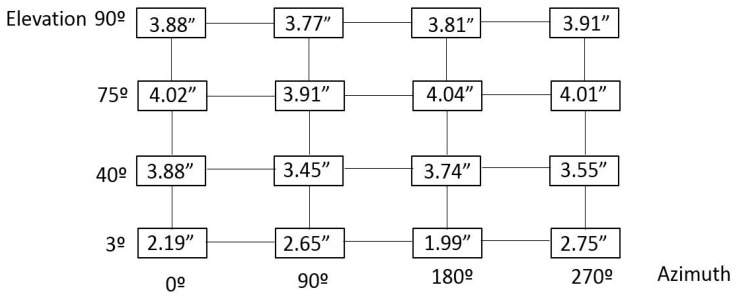
RPE measurement uncertainty results with floor movement consideration (in arcseconds).

After understanding how the floor movement behaved, the RPE simulation model was completed with floor dimensional drift information. Thus, a random floor movement with a Gaussian distribution was applied to each simulation sample by the means of an additional Monte-Carlo simulation process. This meant that a unique 6 degrees of freedom (d.o.f) transformation was applied onto every single point at each simulation sample, which allowed for the simulation of the real behaviour of the floor where the TMA was mounted. Finally, a new RPE measurement simulation was numerically run and realistic uncertainty results for a 95% confidence level (*k* = 2) were achieved, according to the combined post-processing method mentioned previously, which brought a reduction of the measurement uncertainty. The results are shown in Figure 8.

It should be stated that the simulation model only considered temperature for the floor dimensional drift assessment. The effect of the temperature on the laser tracker measurement system itself was not considered. Additionally, the final workplace of the telescope was environmentally stable because the temperature and pressure would be controlled inside the dome.

### 2.5. On-Site Measurement Strategy

After simulating the measurement uncertainty for the RPE verification of the LSST, the results made one thing completely clear: the measurement uncertainty for the metrology network characterization should be kept within 0.1 mm to achieve the RPE measurement uncertainty results between 1 ÷ 2 arcsec (correlation on a stable temperature measurement scenario, e.g., 20 ± 1 °C on the complete LSST volume).

**Figure 9:**
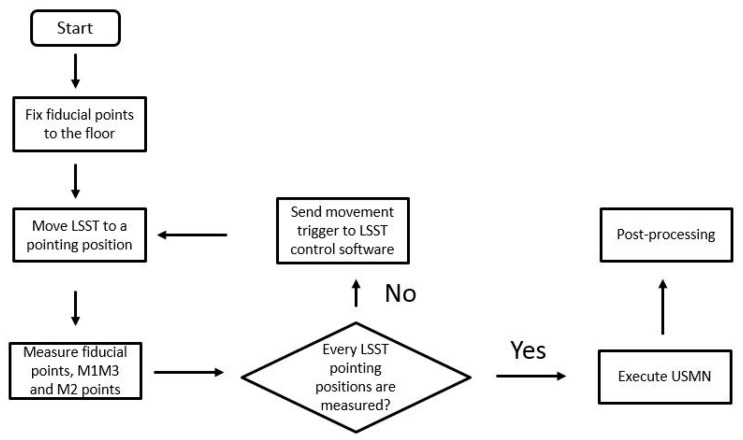
Fully automatic RPE verification procedure flow chart.

At this point, two main limitations were considered for a successful implementation of the suggested new verification method for the RPE assessment: both the laser tracker uncertainty and the temperature effect during the data acquisition period. For the laser tracker uncertainty limitation, the USMN tool was employed within SA software to improve the coordinate uncertainty field computation [18] for the metrology network characterization. For the temperature effect, a fully automatic verification procedure was suggested, to reduce data acquisition time. The laser tracker-based measurement program was interconnected to the LSST control software by the means of a Transmission Control Protocol/Internet Protocol (TCP/IP) in a private connection, and the USMN was applied during the measurement procedure once, so that every fiducial point was measured for every LSST pointing position. The TCP/IP connection permitted the synchronization of the LSST movement with the laser tracker measurement sequence. Figure 9 shows the fully automatic verification pointing measurement procedure for the LSST as a flow chart, where the parallelism measurement between M1M3 and M2 was also considered.

The fully automatic measurement procedure presented in Figure 9 aimed to reduce the RPE measurement down to 60–90 min, and it improves the simulation sequence to one stage.

### 2.6. Validation of Simulated Results—Technological Risk Management

The objective at this point was to validate the developed simulation procedure for the metrology network characterization, considering that its measurement uncertainty should have a maximum of 0.1 mm to achieve the RPE measurement uncertainty results between 1 ÷ 2 arcsec. (correlation on a stable temperature measurement scenario).

**Figure 10:**
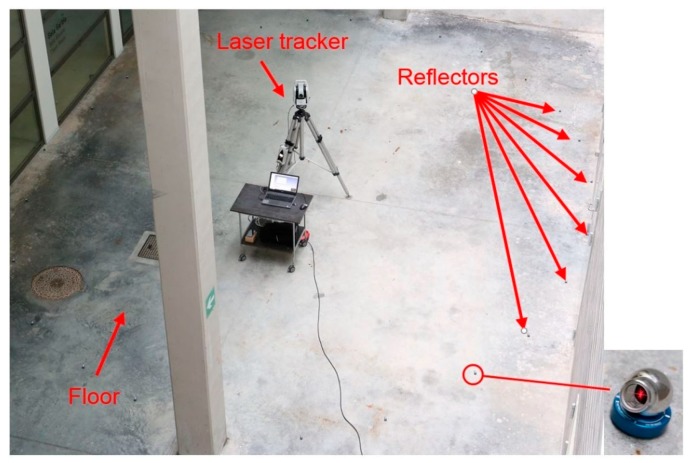
Measurement scenario at IK4-TEKNIKER for simulated results validation.

To do that, the simulation results were compared with real measurement results. A similar shaped 1:2 scale measurement scenario was tested at IK4-TEKNIKER premises, as shown in Figure 10. A Leica AT402 laser tracker has been employed to measure 36 targets distributed on a circular-layout, similar to the real measurement scenario depicted in Figure 1. These targets play the same fiducial role of the metrology network for the LSST measurement scenario.

**Figure 11:**
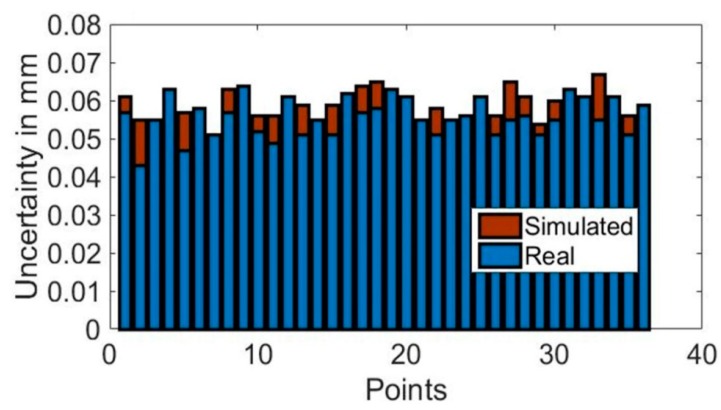
Metrology network characterization validation results.

On the one hand, measurement scenario is simulated with the USMN tool within SA software, with the same simulation code, as described in Section 2.3.1. On the other hand, the measurement scenario was measured 10 times to understand the repeatability contribution of the laser tracker measurement to the obtained uncertainty. After that, simulation-based results are compared to real measurement results on a 1:2 scale LSST measurement scenario. The metrology network characterization results are shown in Figure 11. Here, the combined standard uncertainty value displayed in the vertical axis of the Figure 11 was obtained by summation in the quadrature of each axis uncertainty contribution, obtained by the simulation results. This is defined in Equation (2):

(2) $$U=\sqrt{U_{x}{}^{2}+U_{y}{}^{2}+U_{z}{}^{2}}$$

Experimental test results showed that the simulation-based results and real measurement results were within 0.01 mm in difference, which helps us to understand that the developed measurement procedure tested in the simulation mode resembled its real measurement performance. Thus, the technological risk that was related to the measurement procedure implementation on the real measurement scenario could be reduced and finally managed.

### 2.7. Relative Pointing Error Calibration

Finally, the RPE calibration will be executed on the TMA with the suggested measurement methodology. For that purpose, a minimum of 10 measurements will be executed to understand the LSST positioning repeatability.

When performing the LSST calibration, three main uncertainty contributors were considered for the uncertainty budget, as described in JCGM 100:2008 guide (Evaluation of measurement data—Guide to the expression of uncertainty in measurement) [21]:

- *u_cal_*: standard uncertainty that is associated with the uncertainty of the measurement methodology (measurement technology and measurement procedure). Simulated uncertainties depicted in Figure 6 will be employed for the purpose.
- *u_p_*: standard uncertainty that is associated with the variability of the observed values. It is calculated by the standard deviation of the measured values (*n* = 10).
- *u_b_*: standard uncertainty that is associated with the systematic error of the measurement process for every LSST pointing position.

The expanded measurement uncertainty, U, is calculated for a 95% confidence level (*k* = 2):

(3) $$U=k*\sqrt{u_{cal}{}^{2}+u_{p}{}^{2}+u_{b}{}^{2}}$$

However, if any other source of uncertainty appears during measurement execution, it will be stated in Equation (3).

## 3. 3D Metrology Integration into LSST Structure

Various new-generation large telescope projects in the design or early construction stages are considering using laser trackers as a built-in alignment tool that is available to the telescope control system, and that is integral to the basic operation of the telescope [13]. Laser trackers have been historically employed for optical mirror alignment and engineering tasks, and it is interesting to further investigate the application of on-board laser tracker technology for active telescope alignment. This part of the paper discusses this idea. A metrology simulation tool was presented for the study of the measurement parameters affecting the accuracy of the on-board laser tracker survey and to determine if it was compatible with the required mirror positioning accuracy.

Rakich et al. already tested laser tracker technology on the LBT telescope [13]. They suggest that it is a metrology instrument that is capable of automatically measuring optical element positions with better than 100 µm precision within a spherical volume of 30 m radius centred on the tracker head. They also suggest that the laser tracker is capable of measuring optical component positions during telescope use, with accuracies in the order of 20 microns root mean square (RMS) [13]. These values are considered as a reference for the research work presented in this paper.

**Figure 12:**
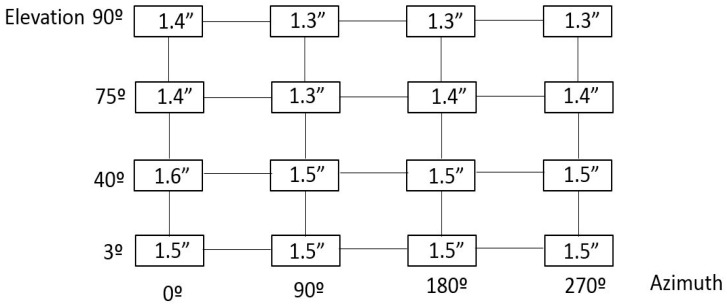
Alignment parallelism uncertainty results under stable floor conditions (in arcseconds).

Regarding the RPE verification exercise, the simulation code was extended to employ laser tracker technology for the measuring of the alignment between the secondary (M2) and the primary/tertiary (M1M3) mirrors as a part of the active alignment system of the LSST (see Figure 1). Thus, four additional points were added to the M2 mirror, similar to the four that were used for the M1M3 definition shown in Figure 1a, and parallelism measurements were executed between the M1M3 and M2 mirrors for every pointing position depicted in Figure 2. Figure 12 shows the alignment parallelism uncertainty results under stable ambient conditions (e.g., 20 ± 1 °C on the complete LSST volume), where the metrology network is fixed and does not change. The results were based on 2 sigma values (95.46% confidence interval) for the mapping matrix defined at Figure 2.

Results in Figure 12 show that alignment parallelism uncertainty results were similar on every pointing position of the LSST, which makes sense because it was a local measurement between the M1M3 and M2 mirrors. In that sense, the next alignment parallelism simulation was executed to guarantee that parallelism did not change with variations of external conditions, and effectively it was a local measurement. Table 1 shows the simulated uncertainty results for the parallelism between M1M3 and M2 mirrors for different values of floor movement.

Results certified that alignment parallelism uncertainty did not change according to the floor variation. As previously commented, this made sense, since the measurements were locally executed, and therefore they did not depend on how the floor performed. The results in Table 1 are based on 2 sigma values (95.46% confidence interval).

The main conclusions that were obtained from the simulation of the active alignment system with an on-board metrology system for the LSST are explained next:

- The measurement was locally executed between the M1M3 and M2 mirrors, so that the results did not depend on the accuracy of the fiducial points fixed to the floor.
- The achievable accuracy with a unique on-board laser tracker and that was centred on the telescope structure, was approximately 1.5 ÷ 1.6 arcsec. Four measuring points were considered respectively in the M1M3 and M2 mirrors.
- The required mirror positioning accuracy was 1 arcsec. It means that the presented measurement strategy was not yet compatible with the required accuracy.

### 3.1. Metrology Simulation Tool

**Figure 13:**
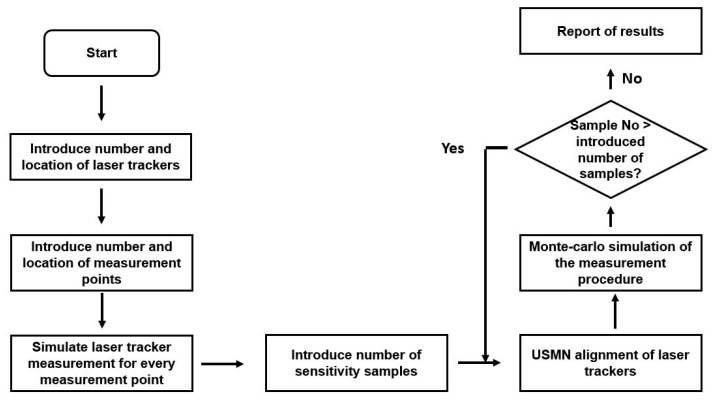
Metrology simulation tool flowchart.

Thanks to the knowledge generated on the RPE simulation model construction where laser tracker technology combined with SA software was employed for a Monte-Carlo simulation of the real measurement scenario, a metrology simulation tool was developed for the feasibility assessment of LSM surveying techniques in the LSST. Some key information such as the number and location of the measurement points, and the number and location of the laser trackers, were introduced into the simulation model to understand how they would affect the achievable measurement accuracy. Output information is useful for understanding the feasibility, the anticipated accuracy limits, constraints, the measurement strategy, or the level of effort for the implementation of the suggested survey strategy. Figure 13 presents the metrology simulation tool flow chart.

At the present stage, the metrology simulation tool simulates the M1M3 and M2 mirrors measurement. As a result, the centroid that defined their position on the LSST reference system was obtained, as well as the normal vector that defined the pointing direction of each mirror and the angle between them, which showed the parallelism between mirrors.

### 3.2. On-Board Metrology Simulation Results

The presented metrology simulation tool also aimed to simulate some measurement scenarios on the LSST to understand the influence of the number of laser trackers and the number of the measurement points on the measurement accuracy. Thus, input variables were defined as:

- The number of on-board laser trackers: from 1 to 4. For a permanent installation of laser trackers into the LSST structure, they were located at the M1M3 mirror level with a 7.400 mm radius.
- Number of measurement points: four points or eight points could be selected to define the geometric plane in each mirror.

The simulation tool numerical output provided the standard deviation of each of the six degrees of freedom of a plane. The translation components define the centroid of the plane, and the rotary components define the normal vector. Thus, the combined standard uncertainty of the centroid were obtained by summation in the quadrature of each axis’ translation uncertainty contribution, according to Equation (2). Similarly, the combined standard uncertainty of the normal vector was obtained by summation in quadrature of each axis’ rotary uncertainty contribution.

**Figure 14:**
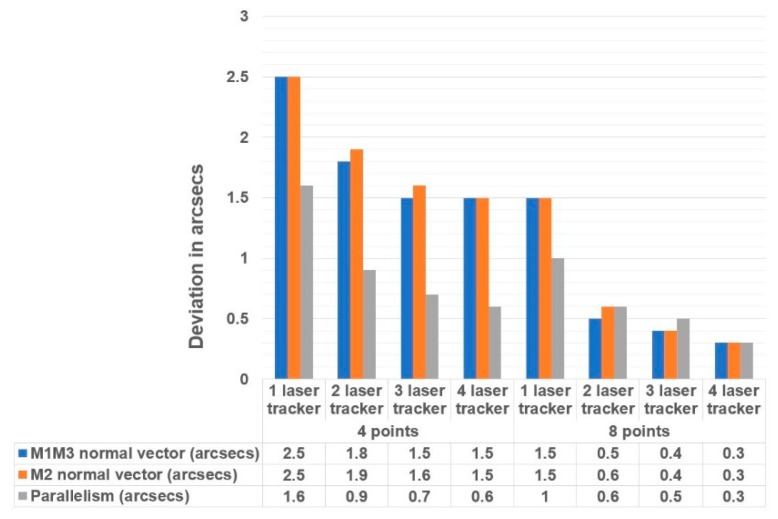
Measurement uncertainty of the normal vector of the M1M3 and M2 mirrors.

**Figure 15:**
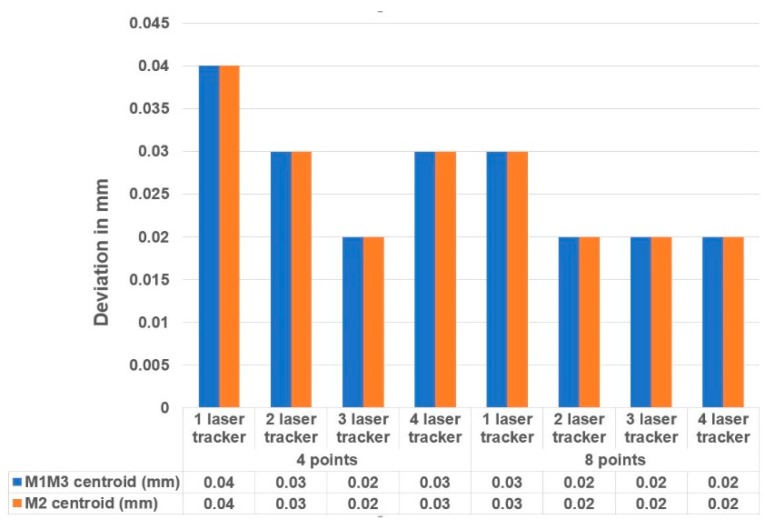
Measurement uncertainty of the centroid of the M1M3 and M2 mirrors.

Figure 14 shows the combined measurement uncertainty of the normal vector of the M1M3 and M2 mirrors, and the parallelism between them. Figure 15 shows the combined measurement uncertainty of the centroid of the M1M3 and M2 mirrors. The results are based on two sigma values (95.46% confidence interval).

The simulation results illustrated that the influence of the number of laser trackers was not as important as the number of measurement points employed on the on-board metrology survey. Similar results have been obtained with a unique laser tracker with eight measurement points, compared to three laser trackers with four measurement points. The best price-performance ratio was achieved with a measurement strategy comprised by two laser trackers and eight measurement points where both the normal vector uncertainty and the centroid uncertainty were compatible with the required mirror accuracy.

Obtained uncertainty results also anticipated the accuracy limits for those LSM survey works on large telescopes. As suggested by Rakich et al., it has been demonstrated that the measurement of optical component positions during telescope use can be accomplished with accuracies in the order of 20 microns (RMS) [13]. Additionally, the required mirror positioning accuracy, which is better than 1 arcsec, can also be obtained with a measurement strategy based upon two on-board laser trackers and eight measurement points.

## 4. Conclusions

A new methodology has been presented and numerically validated for the measurement of the relative pointing error requirement of the TMA subsystem for the LSST. A complete simulation model has been built based upon the Monte-Carlo technique within SA software to anticipate the measurement uncertainty with the suggested methodology. Simulation results show uncertainties better than 5 arcsec for every pointing position within the pointing range of the LSST, which means that the presented methodology is compatible with the RPE requirements, limited to 50 arcsec at a 95% confidence level (*k* = 2). Additionally, a fully automatic RPE verification procedure is presented to reduce the RPE data acquisition down to 60–90 min and, therefore, to reduce the thermal drift of the large-scale measurement scenario.

Regarding the RPE verification exercise, the simulation code has been extended to employ laser tracker technology for the measurement of the alignment between secondary (M2) and primary/tertiary (M1M3) mirrors as a part of the active alignment system of the LSST. Simulated results show that parallelism measurement is a local measurement between the M1M3 and M2 mirrors and therefore the obtained parallelism uncertainty results, better than 1.6 arcsec, can be guaranteed within the pointing range of the LSST.

Based upon the knowledge generated by the RPE simulation model construction, a 3D metrology simulation model has been built to assess the fitness for the purpose of on-board laser tracker technology for performing the alignment of the optical axis and the active telescope alignment. This simulation platform also employs the Monte-Carlo technique to understand how the number and location of the measurement points and laser trackers affect the achievable measurement accuracy. The simulation results show that the best price-performance ratio is achieved with a measurement strategy comprised of two laser trackers and eight measurement points, which is compatible with the required mirror positioning accuracy that is limited to 1 arcsec in the LSST project. This measurement configuration also demonstrates that the measurement of optical component positions during telescope use can be accomplished with accuracies in the order of 20 microns (RMS). Moreover, simulation results conclude that the influence of the number of measurement points is more critical than the number of laser trackers that are employed on the on-board metrology survey.

## 5. Future Work

The future work is focused on executing the RPE verification on the LSST and on validating the simulation results presented in this research article. In addition, it will be interesting to analyse how the RPE verification results can provide compensation values to the kinematic modelling of the TMA. Thus, the TMA pointing error could be corrected if needed.

## Figures and Tables

**Table 1:** Floor movement alignment parallelism simulation (in arcseconds).

Floor Movement (mm)	Parallelism Uncertainty (arcsec)
0.05	1.5
0.1	1.6
0.5	1.5
1	1.6
3	1.5
5	1.5
